# Effects of lethal management on gray wolf pack persistence and reproduction in Wisconsin, USA

**DOI:** 10.1038/s41598-024-60764-6

**Published:** 2024-04-30

**Authors:** Alejandra Zubiria Perez, Kenneth F. Kellner, David M. MacFarland, Jennifer L. Price Tack, David B. Ruid, Glenn E. Stauffer, Jerrold L. Belant

**Affiliations:** 1 Department of Fisheries and Wildlife, Michigan State University, 480 Wilson Road, 17 NR, East Lansing, MI 48824 USA; 2 Office of Applied Science, Wisconsin Department of Natural Resources, Rhinelander, WI 54501 USA; 3 USDA/APHIS/Wildlife Services, Rhinelander, WI 54501 USA

**Keywords:** Anthropogenic mortality, *Canis lupus*, Carnivore management, Howl surveys, Legal harvest, Pack persistence, Conservation biology, Population dynamics

## Abstract

Direct human-caused mortality accounts for about half of all large mammal mortality in North America. For social species like gray wolves (*Canis lupus*), the death of pack members can disrupt pack structure and cause pack dissolution, and mortality of breeding adults or wolves during reproduction and pup-rearing can decrease pup recruitment. We estimated minimum and maximum probability of wolf pack persistence in Wisconsin, USA, during biological years (15 April–14 April) 2011–2019 and evaluated the influence of pack size and legal harvest mortality on pack persistence during 2012–2014. Harvests comprised 75–161 mortalities within 194 monitored packs during 2012–2014, with 56–74% of packs having no wolves harvested each year. As an index of reproduction during 2013–2019, we also estimated the proportion of packs where pups responded to howl surveys. We evaluated the influence of pack size, legal harvest, and agency removal on reproduction during 2013–2015. Annual maximum pack persistence probability was uniformly high (0.95–1.00), and annual minimum pack persistence probability ranged from 0.86–0.98 with a possible decline during years of harvest. Reproduction was similar in years following harvest and agency removal (2013–2015, pup response = 0.27–0.40), and years without harvest or agency removal the year prior (2016–2019, pup response = 0.28–0.66). Pack size had a positive effect on pack persistence and reproduction. Total number of wolf mortalities and number of adult male and females removed did not influence pack persistence or reproduction. We suggest that low per-pack mortality, timing of harvest and agency removal, and harvest characteristics during 2012–2014 supported stable pack persistence and reproduction.

## Introduction

About half of large-vertebrate mortality can be directly attributed to human causes which include vehicle collisions, poaching, agency removal, and legal harvest^1^. Harvest typically occurs during designated periods in fall or winter and can represent over 70% of anthropogenic mortality for mammals^1^, yet some species can sustain high rates of harvest with no effects on population abundance. For example, an increase in immigration and recruitment of cougars (*Puma concolor*) to areas < 1000 km^2^ offset harvest mortality resulting in stable populations^2^. Harvest rates ≤ 29% are sustainable for gray wolves^3,4^, though higher rates can be sustained in some cases^5^. For large carnivores, other sources of anthropogenic mortality account for most deaths where harvest is not allowed^6,7^. Although natural deaths can decrease when anthropogenic mortality increases^5^, human-related mortality can differ from natural death in timing^8,9^ and across sex-age groups^10,11^. Management actions (e.g., harvest quotas and timing of hunting), hunter selection, or individual variation in animal vulnerability can result in anthropogenic mortalities being demographically selective. For example, hunter selection of larger bobcats (*Lynx rufus*) in Wisconsin, USA, resulted in a higher proportion of older, male bobcats harvested^12^. In Sweden, grizzly bears (*Ursus arctos*) with lower activity and that spent more time closer to roads were more susceptible to harvest^13^.

In social species, the loss of one member can reduce the group’s ability to secure resources and reproduce. African elephants (*Loxodonta africana*) exhibited higher physiological stress and reduced reproductive performance in groups when the matriarch was killed^14^. Lion (*Panthera leo*) populations where management removals are common due to livestock depredations experienced reduced reproductive success^15^. However, reduced group size can also lead to greater food availability for remaining members^5^ and more breeding opportunities for subordinates^16^. Group-level responses to mortality can vary with group size and population density^17^ and the demographics of mortality^18^.

Anthropogenic mortality accounted for 61% of gray wolf deaths in studies across North America during 1968–2014^19^. Wolf harvest seasons can overlap with important life history events such as mating^20^, which can affect reproduction and exacerbate the effects of harvest mortality^21^. Gray wolves have complex social dynamics and a hierarchical group structure usually comprising a breeding pair, pups, and possibly non-breeding adults and subadults^5^. The loss of one wolf can lead to pack dissolution, particularly when breeding individuals are removed and pack size is small^18,22^. In stable populations and for larger packs, remaining or immigrating individuals can occupy the role of the removed member and maintain social functions^22^. Breeder loss can also decrease reproduction and recruitment when removal coincides with mating or pup-rearing^21,22^. The loss of adult members or subadults involved in pup rearing can lead to decreased pup survival if fewer members are available to secure food and care for pups^23^. However, multiple subordinate females could breed and increase recruitment the following year if the loss of adult females occurs early in the mating period^16^.

We first examined wolf pack persistence in Wisconsin during the 2011–2019 biological years (15 April–14 April;^24^) by estimating annual maximum and minimum probability of wolf pack persistence. Wolf harvest was allowed during a subset of those years (2012–2014), and we expected probability of pack persistence would be lower during this period due to increased mortality. We then evaluated the effect of pack size and harvest mortality on wolf pack persistence during biological years 2012–2014, predicting that smaller pack size and higher mortality, particularly of adult females, would result in lower probability of pack persistence.

We also examined reproduction in Wisconsin during biological years 2013–2019 by estimating the minimum proportion of wolf packs that reproduced (hereafter ‘reproduction’). We expected that reproduction would be lower in years following harvest and non-harvest lethal management (e.g., depredation control and removal due to safety concerns, disease, or injury; hereafter ‘agency removal’) which occurred during 2012–2015. We evaluated the effect of harvest and agency removal on reproduction in the following springs (i.e., spring 2013–2015) predicting that smaller pack size and higher mortality, particularly of adult females, would result in lower reproduction.

## Methods

### Study area

We studied the gray wolf population in northern and central Wisconsin, USA, where wolves occupy about 91,000 km^2;^^25^. The Wisconsin wolf population is part of the Western Great Lakes Distinct Population Segment which comprises about 4400 wolves in Minnesota, Wisconsin, and Michigan and is connected to larger populations in Canada^26^. The primary prey of wolves in the western Great Lakes Region are white-tailed deer (*Odocoileus virginianus*), which comprise up to 90% of consumed prey biomass, but wolves also feed on beaver (*Castor canadensis*), elk (*Cervus canadensis*), moose (*Alces alces*), and snowshoe hare (*Lepus americanus*;^27^). Other large predators in the area include black bear (*U. americanus*) and coyote (*C. latrans*).

Bounties and unregulated hunting and trapping in Wisconsin since 1839 led to the extirpation of gray wolves by 1960^28^. Gray wolves were first listed as endangered in 1967 under the Endangered Species Preservation Act (ESPA) and were federally protected in 1974 under the Endangered Species Act (ESA;^29^). Wolves began recolonizing Wisconsin in 1975^28^, and by 1999 the Wisconsin–Michigan population had exceeded the federal recovery goal of 100 individuals for 5 consecutive years^30^. Consequently, gray wolves were reclassified as threatened under the ESA in 2003 which allowed Wisconsin to implement lethal removal of wolves involved in conflicts with humans^31^. Gray wolf protection status has since changed ten times through 2022^29^. During our study period, gray wolves within the western Great Lakes were delisted, and hunting, trapping, and lethal agency removal were consequently allowed in Wisconsin, during 28 December 2011–20 February 2015, after which legal actions required reinstatement of ESA protections^26,32^. Western Great Lakes wolves were again delisted on 4 January 2021, but protection under the ESA was reinstated on 10 February 2022^33^. Under ESA protection, lethal control can be used if human safety is a concern (50 CFR 17.21(c)(3)(iv)) but is uncommon in Wisconsin.

### Data

We used data collected annually by the Wisconsin Department of Natural Resources (WDNR) on pack territory boundaries, minimum pack size, and pup responses to howl surveys to estimate pack persistence and index reproduction. We also used mortality data collected by the WDNR and U.S. Department of Agriculture Wildlife Services to assess the impact of legal harvest and agency removal on pack persistence and reproduction (Table A1).

**Figure 1:**
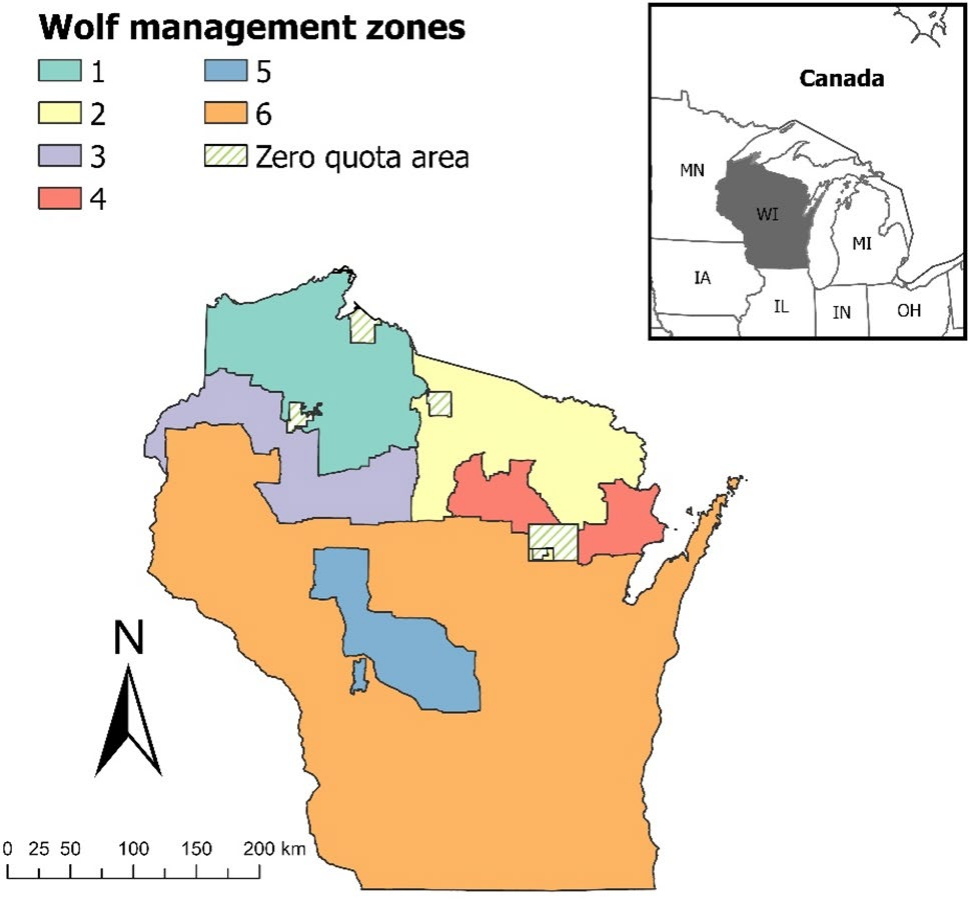
Wolf management zones, Wisconsin (WI), USA, 2012–2023. Zero quota areas represent tribal reservations.

The WDNR monitors wolves annually to estimate population size, pack sizes, and pack territory boundaries within six wolf management zones and tribal reservations (Fig. 1;^24^). Wolves are captured and radio-collared annually by the WDNR. Telemetry locations were used to estimate annual territory boundaries using minimum convex polygons^28,34^, and radio-collared wolves were tracked via aerial surveys each winter to estimate pack size. Aerial and snow track surveys, conducted each winter by WDNR staff, tribal biologists, and volunteers who record locations of all wolf sightings and signs (e.g., tracks, scat) observed, were used to estimate minimum pack size and territory boundaries of packs without collared individuals and to supplement data for packs with monitored individuals^24,28^. Snow track surveys targeted areas with historical wolf presence, reported wolf observations, and highly suitable habitat^28,35^ and consistently covered over 90% of survey blocks^20,24,36–42^. Minimum pack size was estimated using the maximum number of individual track sets located together during a single observation across all surveys and data obtained while tracking radio-collared wolves. Approximate territory boundaries were generated by creating polygons containing telemetry locations, when available, and all locations of sign, tracks, and public sighting reports of wolves within assumed packs and territory boundaries from the prior year if the pack was collared and field sign did not indicate a territory shift^28^. Individual packs were distinguished based on spatial distribution, timing, and directionality of track and sign observations, locations of radiocollared packs, historical pack use, and information on den and rendezvous sites^28^. Annual wolf territory maps and pack counts generated by the WDNR represented pack status during winter of the respective biological year.

Howl surveys to monitor general pack location, group size, and pup presence were conducted primarily from dusk to 0100–0200 h during July–October 2013–2019^43^. Howl surveys comprised surveyors howling from locations within or near delineated pack territories to elicit howl responses. Surveyors attempted to elicit responses about every 2.5 km but sometimes deviated to improve likelihood of response^43^. When a response was heard, the number of responding adults and pups and the estimated distance to and compass azimuth of the response was recorded^43,44^. We used wolf territory boundaries from the previous biological year and distance and direction of howl responses to determine which pack was associated with each response.

We used data from reported wolf mortalities from harvest or agency removal collected by the WDNR and U.S. Department of Agriculture Wildlife Services during 2012**–**2015. Though natural mortality and poaching can be difficult to detect^45^, WDNR obtains coordinates of each harvested wolf from hunters and agency removal is only performed by government agencies which record the location of mortalities. Wolves were identified as male or female and as pup (< 1 year old) or adult (≥ 1 year old). Using coordinates of mortalities, we assigned each wolf mortality to a pack when the mortality occurred within a mapped pack territory during the same or subsequent biological year, or if the mortality was within 3 km of a single pack territory. We selected this 3-km distance as 89% of mortalities were within a territory (60%) or within 3 km of a single pack boundary (29%). We removed 11% of mortalities, of which 2% were within 3 km of two or more territories and we were unable to assign a territory, and 9% were 3–86 km from the nearest known territory. We considered these individuals not associated with a pack and excluded them from analyses. Assessing mortalities within 3 km of a single pack territory can account for extra-territorial movements and seasonal territory shifts^46^ between data collection (winter) and harvest or agency removal (spring and fall of the following year). Wolf mortalities may also occur away from mapped pack territories if hunters used hounds and wolves were pursued from their territory. However, hounds were not used for hunting in 2012^47^ and aided in < 14% and < 4% of harvests in 2013 and 2014, respectively^48,49^, suggesting this occurred infrequently.

### Pack persistence

We evaluated changes in pack persistence during the 2011–2019 biological years. Our data included annual estimates of the number of wolves within each territory as determined by WDNR, which could be zero. We defined pack formation as the first year when at least two wolves were considered to be maintaining a territory. A count of zero wolves for a given territory during years following pack formation could indicate a pack was not detected, not surveyed, or had dissolved, and we were unable to distinguish among these outcomes. Therefore, we estimated two metrics, maximum and minimum pack persistence, which require different assumptions about the interpretation of no wolves observed in a territory during a given year and represent extreme values of estimated pack persistence (Table A1).

To estimate maximum persistence, we assumed that a count of zero wolves in a given territory and year represented a non-detection of a persisting pack, provided wolves were observed in that territory at least one year before and after the year wolves were not detected. This estimate of maximum persistence likely overestimates persistence given that observations of zero wolves in one year, followed by observations > 0 in the following years, could represent a dissolution and new pack formation. However, pack dissolution and territory replacement by a new pack does not usually occur within the same year^22^. To estimate minimum persistence, we assumed packs had dissolved when the annual pack count was zero and considered all counts ≥ 2 in subsequent years to belong to a newly formed pack, following our criteria for pack formation. This estimate is likely an underestimate of pack persistence as some zero counts could be due to methodological issues such as non-detection or lack of survey effort.

We defined the year of pack dissolution as the year following the last non-zero count for maximum and minimum persistence. We estimated probability of maximum and minimum overall annual pack persistence for eight biological years (2011–2019) using a Kaplan–Meier estimator^50^ in package survival^51^ for program R^52^ considering persistence equal to survival in a traditional survival analysis. We calculated the change in probability of pack persistence between consecutive years, which we considered similar if 95% confidence intervals overlapped (Table A2).

We conducted an additional analysis of data from the 2012–2014 biological years when wolves in Wisconsin were not listed under the ESA and harvest and agency removal were allowed^26^. We evaluated the influence of pack size, total number of harvest mortalities, and number of adult males and adult females harvested on maximum and minimum pack persistence using Cox proportional hazard regression models^53^ (Table A2). Because Cox models typically consider death as the event of interest^53^, we considered pack dissolution (i.e., a pack does not persist) as the event of interest, but for consistency we report results in the context of pack persistence (i.e., the opposite of dissolution). Pack size corresponded to the minimum count estimate for each pack at the end of the previous biological year. We calculated total number of harvest mortalities and number of adult females and adult males harvested within each wolf biological year for each pack. We did not analyze agency removals due to insufficient sample size. We used pairwise correlations to test for multicollinearity of variables^54^ and found no correlation (|r|≤ 0.6).

We fit two sets of candidate Cox models, one for minimum persistence and one for maximum persistence. Each set included five models: (1) null model, (2) pack size only, (3) pack size and total number of mortalities, (4) pack size and number of adult male and female mortalities, and (5) pack size, total number of mortalities, and number of adult male and female mortalities. We included random intercepts by pack in all models to account for variation in baseline hazards^21,45^. We used Akaike Information Criterion corrected for small sample sizes (AICc) to rank models and considered models with ∆AICc ≤ 2 of the top-performing model to have substantial support unless additional covariates resulted in the same log-likelihood with no net reduction in AICc^55,56^. We also report coefficient estimates and 95% confidence intervals which we considered non-significant if they overlapped 0. The response variable for these models is pack dissociation but we interpret coefficient estimates in the context of pack persistence, where β < 0 represents a positive and β > 0 a negative relationship between the covariate and pack persistence. We conducted all analyses using package coxme^57^ for program R^52^.

### Reproduction

We used pup response to howl surveys (1 = pups detected, 0 = pups not detected) during biological years 2013–2019 as an index of reproduction. For all packs monitored during howl surveys, we determined whether pups were detected each year regardless of adult detection and calculated the proportion of packs where pups were detected (Table A1).

We further analyzed a subset of data (2013–2015) from biological years following harvest and agency removal when wolves were not listed under the ESA (2012–2014). We used logistic regression to evaluate the influence of pack size, total number of harvest mortalities, and number of adult males and adult females harvested in the prior biological year on probability of reproduction. We fit the same set of five candidate models described above for persistence (Table A2). Agency removal was infrequent compared to harvest, so we assessed its effect on reproduction during biological years 2013–2015 using three models: (1) null model, (2) pack size, and (3) pack size and total number of agency removals. We included random intercepts by pack and biological year in each model. The response variable for these models is pup response where β > 0 represents a positive and β < 0 a negative relationship between the covariate and pup response. We used package lme4^58^ in program R^52^ for analyses and evaluated models as described for pack persistence.

## Results

### Pack persistence

**Figure 2:**
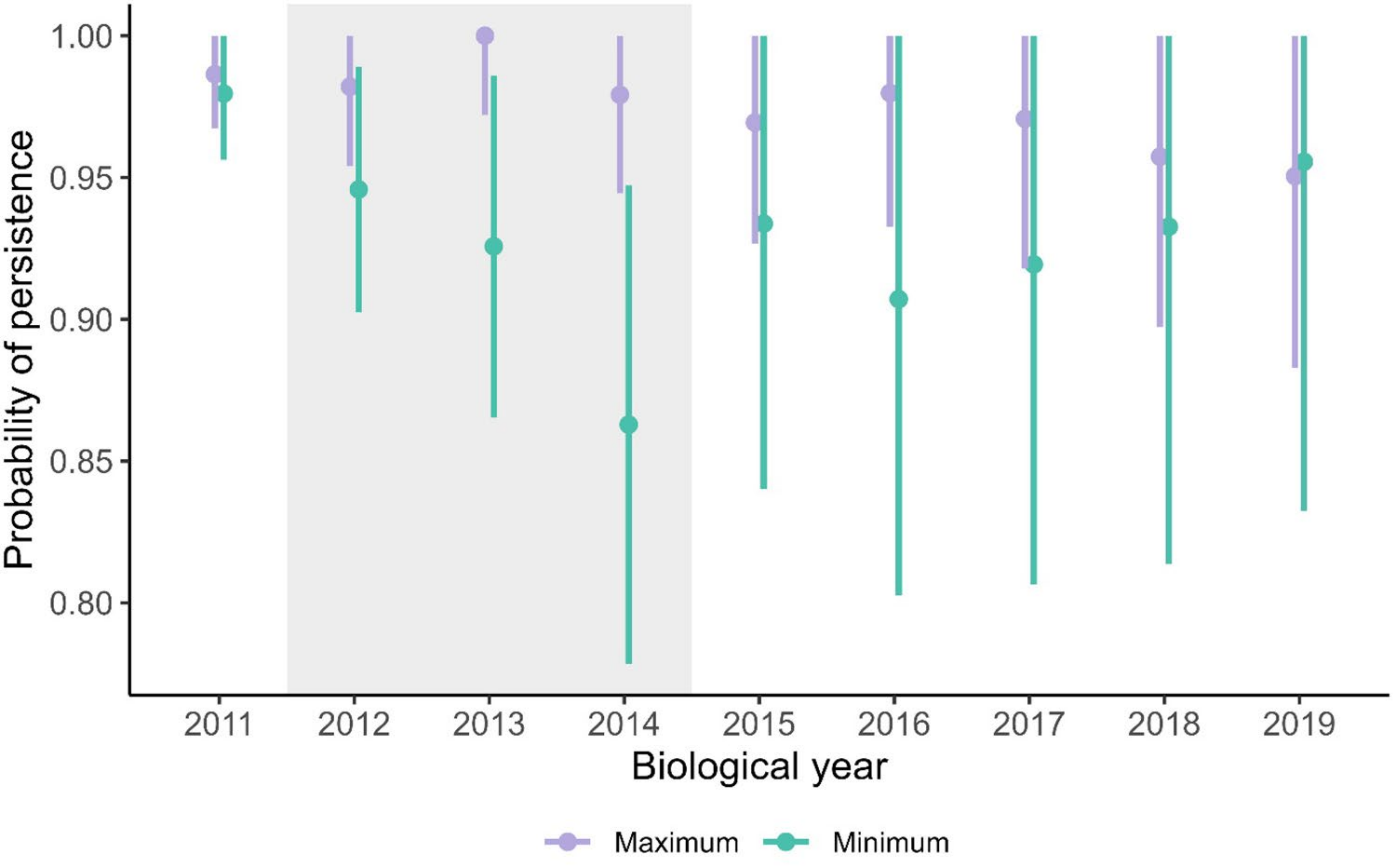
Kaplan–Meier estimates and 95% confidence intervals for change in maximum (purple, n = 236) and minimum (blue, n = 290) wolf pack persistence between consecutive years, Wisconsin, USA, biological years (15 April–14 April) 2011–2019. Shaded area represents years with harvest.

We evaluated maximum persistence for 236 packs during biological years 2011–2019 with median pack size of 3 wolves (range = 1–12). Annual maximum pack persistence probability was 0.95–1.00 (mean = 0.98, SD = 0.02) and was stable across years (Fig. 2). Our estimates of minimum pack persistence included the formation of putative new packs; therefore, we evaluated minimum pack persistence for 290 packs during 2011–2019. Annual minimum pack persistence probability was 0.86–0.98 (mean = 0.93, SD = 0.03; Fig. 2) and was similar across years except 2014 (0.86, 95% CI = 0.78–0.95), which was 0.12 less than the 2011 minimum persistence probability estimate (0.98, 95% CI = 0.96–1.00). Minimum pack persistence probability appeared to decline across years of harvest but stabilized beginning 2015. However, estimates of minimum pack persistence probability were relatively uncertain (Fig. 2).

**Figure 3:**
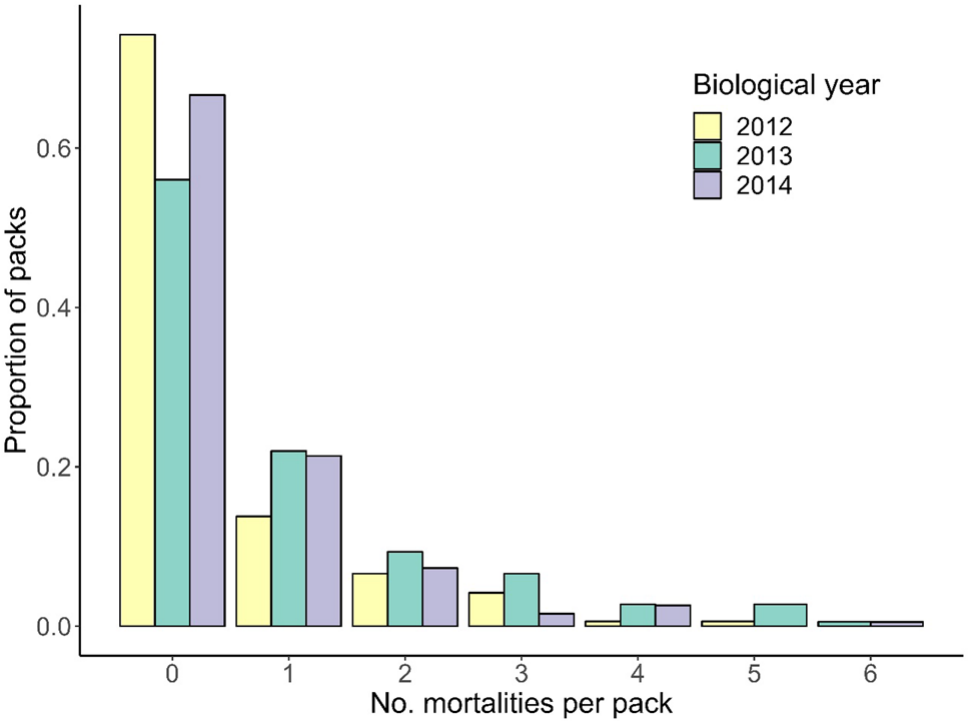
Proportion of wolf packs (n = 194) with harvest mortalities, Wisconsin, USA, biological years (15 April–14 April) 2012–2014.

Harvests comprised 75, 161, and 104 mortalities within 194 monitored packs in the 2012–2014 biological years, respectively. Annual wolf harvest mortalities were zero for 56–74% of packs, one for 14–22% of packs, and two or more for 12–22% of packs (Fig. 3). The top-ranked Cox model for maximum persistence during 2012–2014 included pack size a covariate, but the null model had equivalent support (∆AICc = 0.86, Table 1) and the 95% CI of the coefficient associated with pack size overlapped 0 indicating no effect (β =  − 0.34, CI =  − 0.76–0.08; Table A3). Three maximum pack persistence models failed to converge (Table 1).

**Table 1:** Cox proportional hazard regression model results using Akaike Information Criterion for small sample sizes (AICc) for maximum (n = 194) and minimum (n = 215) wolf pack persistence in response to harvest mortalities (n = 340), Wisconsin, USA, biological years (15 April–14 April) 2012–2014. K = number of parameters including the intercept and one random effect parameter, *w*_*i*_ = model weight, and LL = log likelihood.

Model	K	AICc	∆AICc	*w*_*i*_	LL
Maximum persistence scenario					
PS	3	105.1908	0	0.6064	− 50.5888
Null	2	106.0548	0.8640	0.3936	− 52.0252
PS + TM*					
PS + AM + AF*					
PS + TM + AM + AF*					
Minimum persistence scenario					
PS	3	573.9819	0	0.4948	− 284.9838
PS + TM	4	575.1184	1.1364	0.2836	− 284.5449
PS + TM + AM + AF	6	576.3035	2.3215	0.1527	− 283.1159
PS + AM + AF	5	577.8549	3.8721	0.0689	− 284.9036
Null	2	592.7067	18.7247	0.0001	− 295.3510

PS = pack size, TM = total mortalities, AM = adult male mortalities, AF = adult female mortalities.

*Failed to converge.

The top-ranked model of minimum pack persistence during 2012–2014 included pack size as a covariate and indicated pack persistence increased with increasing pack size (β =  − 0.48, CI =  − 0.72–−0.25; Table A3). The model including both pack size and total number of mortalities had similar support to the top model (Table 1), but the model log-likelihood was only marginally reduced, indicating the additional covariate was uninformative. Furthermore, the effect of total number of mortalities on pack persistence was uncertain (Table A3).

### Reproduction

**Figure 4:**
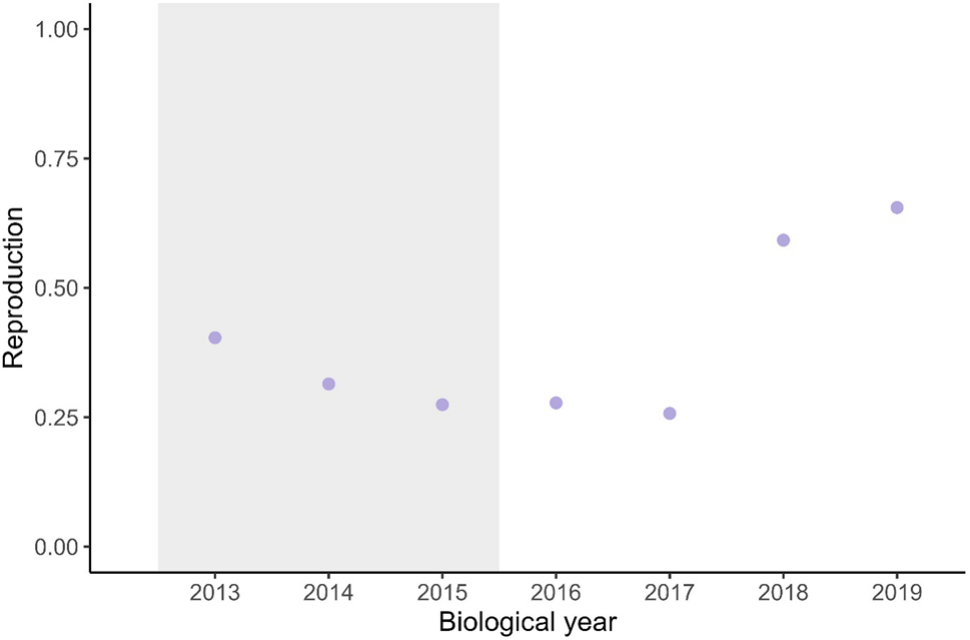
Annual proportion of packs with pups responding to howl surveys (i.e., reproduction) for wolf packs monitored (n = 240) using howl surveys, Wisconsin, USA, biological years (15 April–14 April) 2013–2019. Shaded area represents years following harvest and agency removal.

Howl surveys included 240 wolf packs representing 637 pack-years during 2013–2019 with 1–17 (median = 2) visits per pack annually. For monitored packs, harvest accounted for 50, 92, and 62 mortalities and agency removal accounted for 15, 33, and 26 mortalities in the 2012–2014 biological years, respectively. Reproduction decreased slightly during 2013–2014 (reproduction = 0.31–0.40), was stable during 2015–2017 (reproduction = 0.26–0.28), then increased in 2018–2019 (reproduction = 0.59–0.66; Fig. 4).

Analysis of reproduction including only biological years 2013–2015 comprised 174 packs and 297 pack-years of data. The top–ranked logistic regression model evaluating the effect of harvest on reproduction included pack size only and indicated that reproduction increased with increasing pack size (β = 0.28, 95% CI = 0.08–0.49; Table A4). The model with pack size and total number of mortalities and the model with pack size, number of adult males removed, and number of adult females removed had ∆AICc < 2 (Table 2) but 95% confidence intervals for beta coefficients for total number of mortalities, number of adult males removed, and number of adult females within respective models overlapped 0 indicating uncertain effects on reproduction (Table A4).

**Table 2:** Generalized linear mixed model results using Akaike Information Criterion for small sample sizes (AICc) for reproduction in wolf packs (n = 174) in response to harvest mortalities (n = 204) and agency removals (n = 74), Wisconsin, USA, biological years (15 April–14 April) 2013–2015. K = number of parameters including the intercept and two random effect parameters, *w*_*i*_ = model weight, and LL = log likelihood.

Model	K	AICc	∆AICc	*w*_*i*_	LL
Harvest					
PS	4	370.2388	0	0.3984	− 181.0514
PS + TM	5	370.5149	0.2761	0.3470	− 180.1542
PS + AM + AF	6	371.9607	1.7219	0.1684	− 179.8357
PS + TM + AM + A F	7	373.8439	3.6052	0.0657	− 179.7276
Null	3	376.1651	5.9263	0.0206	− 185.0423
Agency removal					
PS	4	515.3122	0	0.6167	− 253.6061
PS + TM	5	516.3813	1.0691	0. 3608	− 253.1165
Null	3	521.9345	6.6223	0. 0225	− 257.9352

PS = pack size, TM = total mortalities, AM = adult male mortalities, AF = adult female mortalities.

## Discussion

We found no evidence of decreased maximum pack persistence probability during 2012–2014 in the Wisconsin wolf population, while minimum pack persistence probability estimates followed an apparent decreasing trend during those years but were highly uncertain. Our approach to determining pack persistence estimated two extremes. We expect that not all packs with a count of zero in a given year dissolved, nor did all counts of zero represent a non-detection or lack of survey effort. We conclude that actual wolf pack persistence in Wisconsin was likely between our maximum and minimum estimates, and that while the potential decline in minimum pack persistence probability during 2012–2014 could suggest that harvest reduced persistence, high uncertainty in our persistence estimates limited our inference. Further, maximum pack persistence was stable throughout our study period.

Wolf mortality can reduce pack persistence, particularly when breeders are removed from small packs and the population is small (i.e., < 75 wolves^22^). Larger wolf populations as in Wisconsin likely have a larger number of dispersing wolves^22^ that can replace individuals removed from a pack^5^. Although wolf pack sizes in Wisconsin are small (mean minimum pack size in our study = 3.36), per-pack harvest mortalities during our study were low, with more than half of packs each year having no known harvest mortalities. We were unable to account for natural and other causes of human-caused mortality but assumed these were similar across our treatments. It is possible that per-pack harvest rates in Wisconsin were low enough to preclude any negative impacts of mortality or that lost wolves were replaced by dispersers before pack dissolution.

Minimum pack persistence probability in Wisconsin increased with increasing pack size but was not influenced by total number of wolves harvested or number of adults removed. Pack size did not influence maximum probability of persistence, which was uniformly high. Our results showing a positive relationship between pack size and minimum persistence probability support previous documented effects of pack size on pack dynamics, such as larger packs having more individuals available to defend territories, secure food, and aid in pup-rearing^5^ and being less likely to dissolve following wolf mortality^18,22^. However, even larger packs are more likely to dissolve when breeding wolves or adults involved in pup-rearing are removed, with breeder loss preceding 85% of pack dissolutions^22^. We were unable to differentiate breeding from non-breeding adults but expect at least some adults removed were breeders, and assumed all adults contributed to hunting or pup-rearing given wolf eusociality^5^. Pack dissolution is more likely when both breeders are lost^18,22^, which was unlikely in our study since per-pack mortality was low and harvests were pup-biased in 2013 and 2014 (but not 2012; Table A5), suggesting the proportion of breeding individuals lost also was low.

We found no evidence of decreased reproduction in years following harvests (2013–2015) in the Wisconsin wolf population. Harvest may affect reproduction differently if it occurs during the wolf mating period, such as the season in Wisconsin when 218 wolves were harvested during February 22–24 2021^59^. Harvests in Wisconsin during 2012–2014 occurred in October–December and 98% of agency removals occurred from 15 April to November. Only one agency removal occurred during the mating period (late January–early March^31^), and seven mortalities occurred during the gestation period (early March–early April^31^). Reproduction can decline the following year if harvest occurs during the mating period^21^, particularly if mortalities are within three months before mating and breeders are lost^22^. Although agency removal during our study was adult-biased (Table A5), breeder replacement in large populations occurs quickly leading to reproduction within one year^22^. We suggest the low per-pack mortality that did not overlap the mating period and the large population size contributed to stable reproduction rates in years following harvest and agency removal in Wisconsin. However, we did not consider the effects of mortality on litter size or pup survival though harvest can reduce pack size and pup survival due to the loss of individuals contributing to hunting and territory maintenance^23^.

Reproduction was more likely for larger packs which contain more adult wolves that contribute to reproduction success^22^ but was not influenced by harvest mortality or agency removal. Breeder turnover can decrease reproductive success^22^, but within-pack shifts in social hierarchies can increase reproduction^16^. Harvests in Wisconsin during 2012–2014 could have resulted in similar or increased reproduction if the loss of a breeding female allowed for multiple non-breeding females to reproduce^23^.

We presume that the minimum size of the Wisconsin wolf population during years with harvest (660–834 individuals) and connectivity with wolf populations in Minnesota, Michigan, and Ontario^60^ helped maintain pack dynamics through replacement of removed individuals with dispersers. We were unable to consider the spatial distribution of wolf harvests though harvest quotas and harvest rates in Wisconsin differ among management zones^20,21,40,41^. We infer pack persistence and reproduction would remain stable if similar harvest rates were implemented outside of the mating period and harvest demographics are similar to the 2012–2014 harvest seasons. However, wolf responses to human-caused mortality are complex and demonstrate the importance of considering population size and demographics, timing, and magnitude of mortalities when implementing harvests and agency removal.

### Supplementary Information

Supplementary Information.

## Data Availability

Data are held by the Wisconsin Department of Natural Resources and represent a protected data set. For data permissions, please contact the Wisconsin Department of Natural Resources.
