# Supplementary material for: Effects of lethal management on gray wolf pack persistence and reproduction in Wisconsin, USA
Source: Sci Rep. 2024 Apr 30;14:9895. doi: 10.1038/s41598-024-60764-6 (PMC11061146; doi:10.1038/s41598-024-60764-6)
Supplement: Supplementary file 1 — Supplementary Information. [file 41598_2024_60764_MOESM1_ESM.docx]

**Table A1.** Explanatory and response variables used in analyses. Data from the Wisconsin Department of Natural Resources (WDNR) and U.S. Department of Agriculture (USDA).

| **Explanatory variables** | | | |
| --- | --- | --- | --- |
|  | Data used | Source | Description |
| Pack size | Minimum pack size | WDNR | Minimum pack size as estimated by WDNR |
| Harvest mortality | Pack territory boundaries  Harvest reports | WDNR | Pack boundaries as estimated by WDNR. Each mortality was assigned to a pack if it occurred within a mapped pack territory during the same or subsequent biological year, or if the mortality was within 3 km of a single pack territory |
| Agency removal | Pack territory boundaries  Agency removal reports | WDNR  USDA | Pack boundaries as estimated by WDNR. Each mortality was assigned to a pack if it occurred within a mapped pack territory during the same or subsequent biological year, or if the mortality was within 3 km of a single pack territory |
| **Response variables** | | | |
|  | Data used | Source | Description |
| Maximum  pack persistence | Pack size | WDNR | Assumed pack counts of zero wolves in a given territory and year represented a non-detection |
| Minimum pack persistence | Pack size | WDNR | Assumed packs had dissolved when the annual pack count was zero and assigned all counts ≥2 in subsequent years as belonging to a new pack |
| Reproduction | Pack territory boundaries  Howl survey responses | WDNR | Determined which pack was associated with each howl response using wolf territory boundaries (as estimated by WDNR) from the previous biological year and distance and direction of howl responses. Reproduction = proportion of packs where pups were detected during a howl survey |

**Table A2.** Summary of analyses for evaluating changes in wolf pack persistence and reproduction, 2011–2019, and impacts of harvest mortality or agency removal on pack persistence, 2012–2014, and reproduction, 2013–2015, Wisconsin, USA. Biological years comprise 15 April–14 April. PS = pack size, TM = total mortalities, AM = adult male mortalities, AF = adult female mortalities.

CI = confidence interval.

|  | **Biological years** | **Response variables** | **Explanatory variables** | **Method** | **No. models** | **Statistical significance** |
| --- | --- | --- | --- | --- | --- | --- |
| Changes in maximum pack persistence | 2011–2019 | Maximum pack persistence |  | Kaplan-Meier estimator | 1 | 95% CI overlap |
| Changes in minimum pack persistence | 2011–2019 | Minimum pack persistence |  | Kaplan-Meier estimator | 1 | 95% CI overlap |
| Impacts of pack size and harvest on maximum pack persistence | 2012–2014 | Maximum pack persistence | PS, TM, AF, AM | Cox-proportional hazard models | 5 | 95% CI of coefficient estimate overlaps with zero |
| Impacts of pack size and harvest on minimum pack persistence | 2012–2014 | Minimum pack persistence | PS, TM, AF, AM | Cox-proportional hazard models | 5 | 95% CI of coefficient estimate overlaps with zero |
| Changes in reproduction | 2011–2019 | Reproduction |  |  |  | Visual comparison |
| Impacts of pack size and harvest on reproduction | 2013–2015 | Reproduction | PS, TM, AF, AM | Binomial generalized linear mixed models | 5 | 95% CI of coefficient estimate overlaps with zero |
| Impacts of pack size and agency removal on reproduction | 2013–2015 | Reproduction | PS, TM | Binomial generalized linear mixed models | 3 | 95% CI of coefficient estimate overlaps with zero |

**Table A3.** Beta coefficient estimates with 95% confidence intervals (CI) and P values for Cox proportional hazard regression models within 2 ∆AICc of best-supported model for evaluating scenarios of maximum (n = 194) and minimum (n = 215) wolf pack persistence probability in response to harvest mortalities (n = 340), Wisconsin, USA, biological years (15 April–14 April) 2012–2014. Because the Cox model evaluates the complement of persistence probability (i.e., pack dissolution), beta parameters less than zero represent a positive influence on pack persistence, and vice versa.

| **Maximum persistence scenario** | | | | | | | |
| --- | --- | --- | --- | --- | --- | --- | --- |
| Predictor | PS | | |  |  |  |  |
|  | β | 95% CI | P |  |  |  |  |
| PS | -0.34 | -0.76–0.08 | 0.110 |  |  |  |  |
| **Minimum persistence scenario** | | | | | | | |
|  | PS | | |  | PS+TM | | |
|  | β | 95% CI | P |  | β | 95% CI | P |
| PS | -0.48 | -0.72– -0.25 | < 0.001 |  | -0.47 | -0.72– -0.25 | < 0.001 |
| TM |  |  |  |  | -0.14 | -0.45–0.17 | 0.367 |
| PS = pack size, TM = total mortalities | | | | | | | |

**Table A4.** Beta coefficient estimates with 95% confidence intervals (CI) and P values for generalized linear mixed models within 2 ∆AICc of best-supported model for estimating reproduction in wolf packs (n = 174) in response to harvest mortalities (n = 204) and agency removals (n = 74), Wisconsin, USA, biological years (15 April–14 April) 2013–2015. Beta coefficients represent a positive or negative effect on persistence if greater or less than zero, respectively.

| **Harvest** | | | | | | | | | | | |
| --- | --- | --- | --- | --- | --- | --- | --- | --- | --- | --- | --- |
|  | PS | | |  | PS+TM | | |  | PS+AM+AF | | |
| Predictor | β | CI | P |  | β | CI | P |  | β | CI | P |
| PS | 0.28 | 0.08–0.49 | 0.007 |  | 0.28 | 0.08–0.48 | 0.007 |  | 0.28 | 0.08–0.48 | **0.006** |
| TM |  |  |  |  | 0.14 | -0.08–0.45 | 0.176 |  |  |  |  |
| AM |  |  |  |  |  |  |  |  | 0.53 | -0.16–1.23 | 0.133 |
| AF |  |  |  |  |  |  |  |  | 0.07 | -0.64–0.77 | 0.856 |
| **Agency removal** | | | | | | | | | | | |
| PS | 0.24 | 0.08–0.40 | 0.004 |  | 0.23 | 0.07–0.40 | 0.005 |  |  |  |  |
| TM |  |  |  |  | 0.12 | -0.12–0.36 | 0.325 |  |  |  |  |
| PS = pack size, TM = total mortalities, AM = adult male mortalities, AF = adult female mortalities | | | | | | | | | | | |

**Table A5.** Number (%) of wolf harvest mortalities and agency removals by sex and age class reported by the Wisconsin Department of Natural Resources, Wisconsin, USA, biological years (15 April–14 April) 2011–2015.

| **Harvest** | | | | | | | |
| --- | --- | --- | --- | --- | --- | --- | --- |
| Biological year | Female adult | Female pup | Female unknown | Male adult | Male pup | Male unknown | Total |
| 2011 | 0 | 0 | 0 | 0 | 0 | 0 | 0 |
| 2012 | 18 (15.4) | 27 (23.1) | 2 (1.7) | 37 (31.6) | 31 (26.5) | 2 (1.7) | 117 |
| 2013 | 54 (21.0) | 68 (26.5) | 1 (0.4) | 56 (21.8) | 75 (29.2) | 3 (1.2) | 257 |
| 2014 | 19 (12.3) | 42 (27.3) | 6 (3.9) | 34 (22.1) | 48 (31.2) | 5 (3.2) | 154 |
| 2015 | 0 | 0 | 0 | 0 | 0 | 0 | 0 |
| Total | 96 (17.5) | 137 (24.9) | 15 (2.7) | 132 (24.0) | 152 (27.6) | 18 (3.3) | 550 |
| **Agency removal** | | | | | | | |
| 2011 | 3 (75.0) | 0 | 0 | 1 (25.0) | 0 | 0 | 4 |
| 2012 | 22 (34.4) | 5 (7.8) | 0 | 30 (46.9) | 7 (10.9) | 0 | 64 |
| 2013 | 25 (37.9) | 12 (18.2) | 1 (1.5) | 20 (30.3) | 7 (10.6) | 1 (1.5) | 66 |
| 2014 | 13 (38.2) | 5 (14.7) | 0 | 12 (35.3) | 3 (8.8) | 1 (2.9) | 34 |
| 2015 | 1 (100.0) | 0 | 0 | 0 | 0 | 0 | 1 |
| Total | 61 (36.3) | 22 (13.1) | 3 (1.8) | 63 (37.5) | 17 (10.1) | 2 (1.2) | 168 |
